# Biotransformation of ferulic acid to vanillin in the packed bed-stirred fermentors

**DOI:** 10.1038/srep34644

**Published:** 2016-10-06

**Authors:** Lei Yan, Peng Chen, Shuang Zhang, Suyue Li, Xiaojuan Yan, Ningbo Wang, Ning Liang, Hongyu Li

**Affiliations:** 1 College of Life Science and Technology, Heilongjiang Bayi Agricultural University, Xinfeng Road No.5, Daqing, 163319, P.R. China; 2 School of Pharmacy, Lanzhou University, Donggang West Road No. 199, Lanzhou, 730020, P.R. China; 3 Gansu Institute of Business and Technology, Yannan Road No. 449, Lanzhou, 730010, P.R. China

## Abstract

We performed the biotransformation of ferulic acid to vanillin using *Bacillus subtilis* (*B. subtilis*) in the stirring packed-bed reactors filled with carbon fiber textiles (CFT). Scanning electron microscope (SEM), HPLC, qRT-PCR and ATP assay indicated that vanillin biotransformation is tightly related to cell growth, cellar activity and the extent of biofilm formation. The biotransformation was affected by hydraulic retention time (HRT), temperature, initial pH, stirring speed and ferulic acid concentration, and the maximum vanillin production was obtained at 20 h, 35 °C, 9.0, 200 rpm, 1.5 g/L, respectively. Repeated batch biotransformation performed under this optimized condition showed that the maximum productivity (0.047 g/L/h) and molar yield (60.43%) achieved in immobilized cell system were 1.84 and 3.61 folds higher than those achieved in free cell system. Therefore, the stirring reactor packed with CFT carrier biofilm formed by *B. subtilis* represented a valid biocatalytic system for the production of vanillin.

Vanillin (4-hydroxy-3-methoxybenzaldehyde), the major compound of vanilla flavor, is widely used in foods, beverages, perfumes, and pharmaceuticals^1^. It can be produced by many technologies including chemical synthesis, hydrolysis and biotransformation^2^. Among them, biotransformation is believed to be most promising eco-friendly method because it can meet the rising demand for healthy and natural vanillin^1^,^2^. Many efforts have been made to biotransform vanillin using natural abundant substrates including lignin, phenolic stilbenes, isoeugenol, eugenol, ferulic acid, vanillic acid, sugars, aromatic amino acids creosol, vanillyl amine, and waste residues^1^.

As one of the most excellent precursors, ferulic acid is a nearly ubiquitous, readily available substance and can be naturally released by a combination of physical and enzymatic processing^3^. Biotransformation of ferulic acid to vanillin have been assessed using various microorganisms, including *Rhodococcus* ssp., *Actinomycetes* spp., *Corynebacterium glutamicum*, *Saccharomyces cerevisiae*, *Rhodotorula rubra*, *Debaryomyces hanseni*, *Halomonas elongata*, *Schizophyllum commune*, *Bacillus licheniformis*, *Bacillus coagulans*, *Bacillus subtilis*, *Pycnoporus cinnabarinus* CGMCC1115, *Pseudomonas* sp. EF3, *Pseudomonas fluorescens*, *Pseudomonas putida*, *Escherichia coli* JM109/pBB1, *Aspergillus niger* CGMCC0774, Lactic acid bacteria, *Amycolatopsis* sp. HR167, *Streptomyces* sp. V-1, *Streptomyces setonii* ATCC39116, *Streptomyces sannanensis*, *Streptomyces halstedii* GE107678, and genetically engineered microbes^1^,^2^,^3^. However, vanillin yield was low mostly due to the inhibitory effect of vanillin on free microbial cells and/or the genetic instability of the recombinant strains during the transformation^2^. Additionally, expensive microbial culture, long-time operation, and complicated following treatments would lead to high cost of biotransformation using free microorganisms^4^.

Immobilization of microbial cells has been attracting worldwide attention for their excellent biological compatibility, high cell concentrations, storage and operational stabilities and the tolerance against harsh conditions^4^. Among immobilization carriers, CFT are regarded as effective microbial cell attached materials because of their surface-hydrophobicity and porous character^5^. CFT have been widely applied in the field of biocatalysts and have proved beneficial for successful operation at high substrate concentration and biotransformation of artificial liquid medium^6^. However, there is a remarkable lack of information about the biotransformation of ferulic acid to vanillin in the stirring reactors packed with CFT.

In the present paper, the production of vanillin from ferulic acid by *B. subtilis* using stirring fixed-CFT reactor was reported for the first time. SEM, HPLC, qRT-PCR and ATP assay were used to investigate the time course of transformation in relation to biofilm formation, vanillin titer in broth, cell growth and cellar activity during immobilizing, respectively. The influences of various parameters, including HRT, temperature, initial pH, stirring speed, and ferulic acid concentration, on vanillin yield in this type of reactor were investigated. The repeated batch biotransformation using immobilized cells or free cells was also performed.

## Results and Discussion

**Figure 1:**
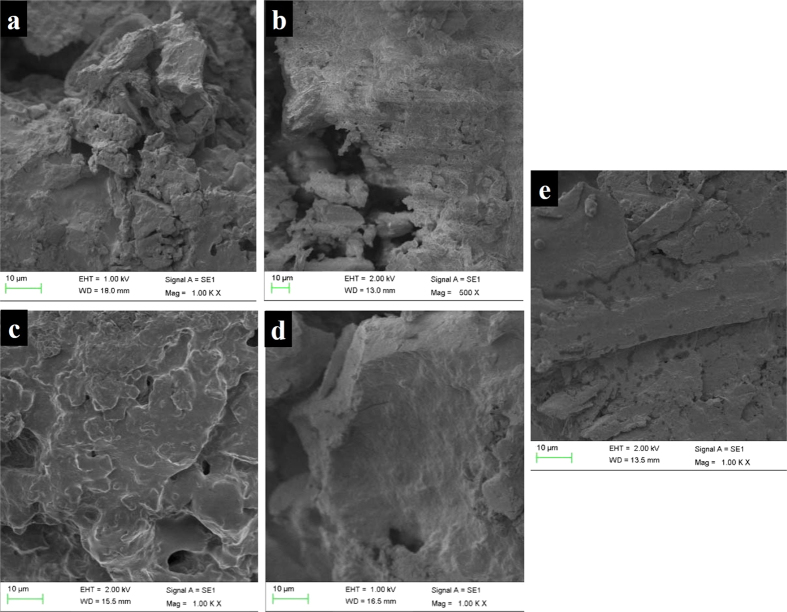
Time course of the extent of biofilm formation on CFT carrier during immobilization using SEM (**a**: 0 h, **b**: 10 h, **c**: 20 h, **d**: 30 h, **e**: 40 h).

The sequence of biofilm formation on CFT carrier in different time intervals (0, 10, 20, 30 and 40 h) could be clearly seen in SEM images (Fig. 1a–e). It can be observed that the CFT carrier at 0 h has a furrowed surface with interstices, pleads and cavities, quite suitable for colonization and apparently offered a larger surface area (Fig. 1a). After 10 h of continuous operation of the packed bed-stirred fermentor, bacterial cells appeared to penetrate into the CFT carrier as shown in Fig. 1b. The co-existence of immobilized- and suspended-growth profile provided the complex hydrodynamic and substrates uptaking circumstances, resulted in overgrowth of bacteria on the interior and surface of CFT carrier^7^. Some holes and channels on CFT carrier surface indicated biofilm formation and extracellular polymeric substances (EPS) production^8^. The EPS play an important role in the immobilization of cells to the carrier, positively correlated with the biofilm development and possibly by protecting the individual cells in the biofilm against detrimental environment^9^. After 20 h, the biofilm structure became denser and thicker (Fig. 1c). It also can be seen from Fig. 1c that CFT carriers were colonized almost exclusively in crevices and pleats. After 30 h, the pleats and some holes were fully colonized showing a dense biofilm, as can be seen in Fig. 1d. The biofilm showed several clearly different layers. This might be attributable to the presence of an area with a lower number of cells and probably more EPS. SEM were taken after 40 h of operation and showed the CFT carrier was completely covered with biofilm (Fig. 1e).

**Figure 2:**
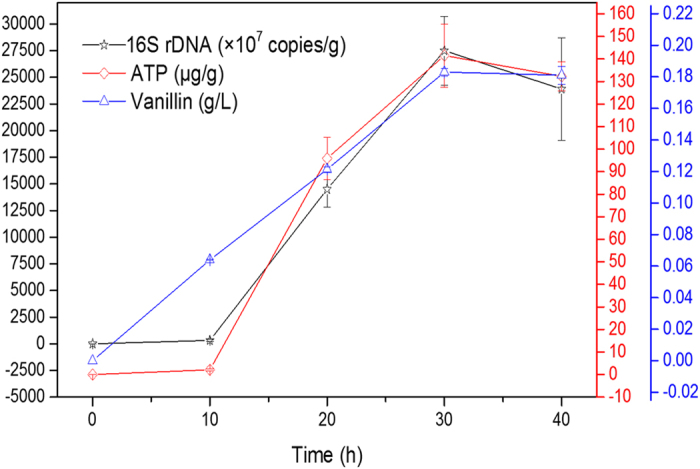
Time course of vanillin concentration, 16S rDNA copy number and ATP content during immobilizing.

To understand the time course of transformation in relation to immobilization and biofilm formation, the vanillin concentration in fermented broth, the cell growth and cellular activity in CFT carrier were monitored during immobilizing. The qRT-PCR was able to measure growth of *B. subtilis* in terms of the 16S rDNA gene number. ATP is present in all living microorganisms as the basic energy molecule, it could be applied to determine cellular activity and measure active biomass^10^,^11^. As shown in Fig. 2, the cell numbers of *B. subtilis* varied in a big range of 7.90 × 10^6^–2.75 × 10^11^ copies/g. It appears that *B. subtilis* had a lag phase at beginning of immobilization. *B. subtilis* attaching to CFT carrier need to acclimate to immobilized medium and carrier niche by incubation for 10 h after inoculation. It can be observered that the amount of *B. subtilis* rapidly increased from 3.19 × 10^9^ copies/g at 10 h to the maximum value at 30 h, and then decreased to 2.39 × 10^11^ copies/g (Fig. 2). A similar trend was observed in time course of ATP concentration (Fig. 2). These indicated that *B. subtilis* successfully immobilized, grown, maintained cellar activity, and formed biofilm on CFT carrier within 40 h. For *B. subtilis*, the amount of ATP is 1.00 × 10^−8^–6.00 × 10^−8^ μg/cell, and the number of 16S rDNA genes per genome is reported to be 9 or probably 10 12. Accordingly, values of cell number obtained by ATP assay were lower when compared to qRT-PCR values. The main reason for this discrepancy was that the qRT-PCR values included potentially extracellular DNA, DNA from viable and non-viable cells, whereas ATP values only contained viable cells.

Concerning vanillin concentration, the value increased from 0 to 0.18 g/L during immobilizing. The increase pattern of vanillin concentration was similar to cell growth and cellar activity before hour 30 (Fig. 2). This suggested that the immobilized cells on CFT carrier could successfully and efficiently transform ferulic acid to vanillin. After hour 30, vanillin concentration maintained stable while cell growth and cellar activity decreased (Fig. 2). This may indicated that, the ferulic acid exhausted and transformed to vanillin within 30 h, leading to termination of vanillin transformation and decline of cell numbers and ATP content. These results suggested that vanillin production is closely related to the growth and activity of cells on CFT carrier biofilm.

Batch experiments were performed at various HRTs (10, 20, 30, and 40 h) to assess the effect of HRT on the vanillin molar yield. Other conditions such as temperature, initial pH, stirring speed and ferulic acid concentration were kept constant at 35 °C, 9.0, 200 rpm and 1.5 g/L, respectively.

**Figure 3:**
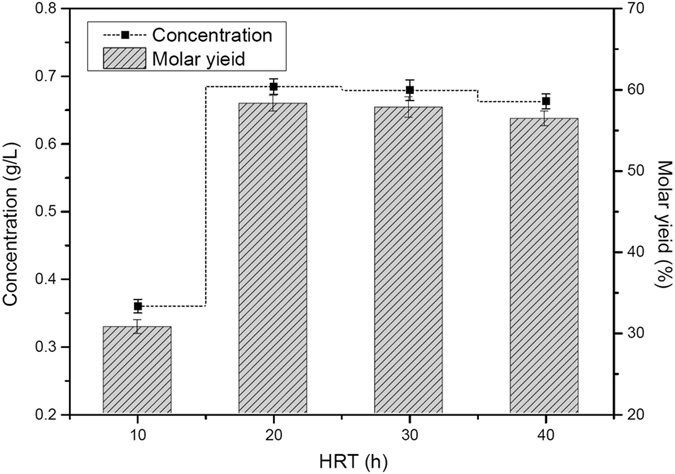
Effect of HRT on the production of vanillin.

Figure 3 shows that the maximal production performance, as peak vanillin molar yield of 58.26% at HRT of 20 h. It can be observed that the vanillin concentration in reactor increased significantly from 10 h HRT (0.36 g/L) to 20 h HRT (0.68 g/L), however, decreased to 0.67 g/L at 30 h HRT. Lowering HRT could lead to a decrease in biotransformation due to the availability of less substrate frequently to microorganisms^13^. It has been reported that highering HRT could improve the production performance because more substrate was solubilized and consumed under the longer HRTs^14^. However, it would result in the high cellular toxicity of vanillin and further degradation of vanillin^13^. It also can be seen form Fig. 3 that the vanillin molar yield declined continually when HRT was over 20 h. This was attributed to the limitation of bacterial growth by the low substrate concentration supplied at higher HRTs. Thus, shortening the HRT to 20 h was sufficient to obtain the highest vanillin molar yield and reduce the product inhibition in biotransformation.

As an important factor in fermentation, temperature has great effect on the bacterial growth, the product generation, and the rheological properties of fermentation broth^15^. To investigate the influence of temperature on biotransformation, the incubation temperature was varied at 25, 30, 35, and 40 °C. The HRT, initial pH, stirring speed and ferulic acid concentration were fixed at 20 h, 9.0, 200 rpm and 1.5 g/L, respectively.

**Figure 4:**
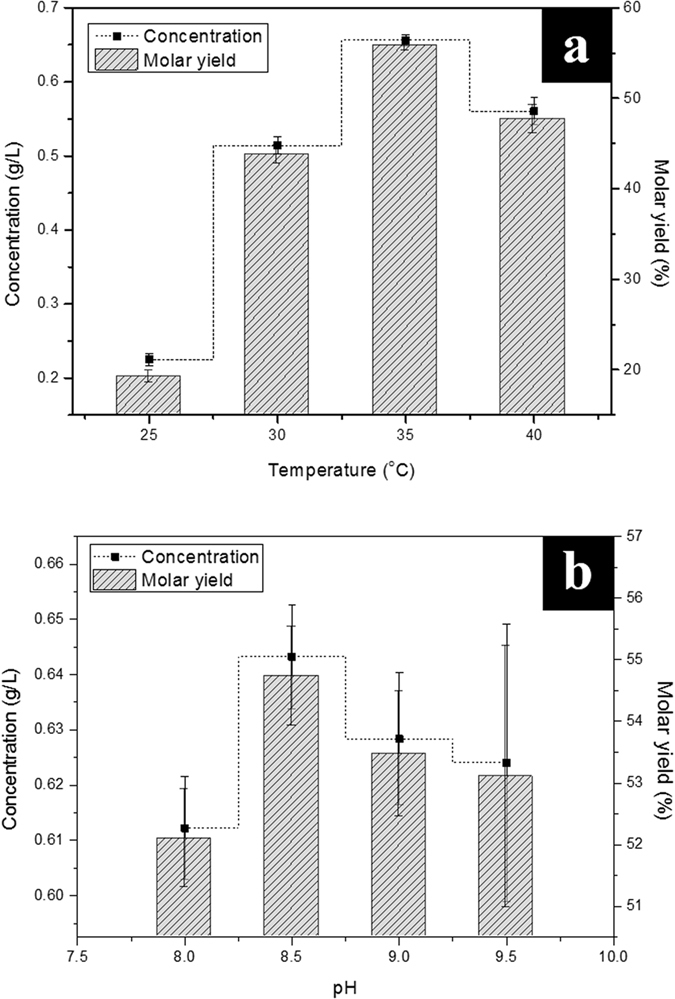
Effect of temperature (**a**) and pH (**b**) on the production of vanillin.

As illustrated in Fig. 4a, a great difference in the production of vanillin was observed when biotransformation was performed at different temperatures. It can be seen that the production of vanillin was sharply increased with the increase of temperature from 25 to 35 °C. However, further increase of temperature resulted in a sharp decrease in the yield of vanillin. The maximum amount of vanillin production was observed at 35 °C and 0.66 g/L vanillin was produced with the molar yield of 55.83% (Fig. 4a).

As we all know, mesophilic temperature fermentation not only reduce the energy consumption for heating and cooling, but also maintains the stability of substrate^16^. Generally, *Bacillus* species have an optimal fermentation temperature range of 30–40 °C to produce products^15^. Only 0.23 g/L vanillin was produced with the molar yield of 19.13% at 25 °C. The primary reason might be that the exchange of nutrients and the diffusion rate of dissolved gases were greatly retarded at low temperature conditions^17^. Additionally, low temperature could negatively influence bacterial growth and biofilm formation in CFT carrier. The elevating temperature could significantly improve the vanillin production due to several reasons. Firstly, the escalating temperature can change the physiological activity of cells, impact the EPS production and change the surface charge of cells^18^,^19^. These would promote the attachment of cells and the biofilm formation during biotransformation. It has been reported that even a small increase in temperature increases the biofilm thickness significantly^20^. Secondly, increasing temperature may influence the secretion and activity of enzyme related to vanillin production such as decarboxylase^1^,^15^. Lastly, escalations in temperature could increase substrate solubility, improve mass transfer due to increased diffusion rates and declined viscosity^15^. It has been stated that a higher temperature would lead to a higher collision rate and higher average kinetic energy of molecules^20^.

It was observed that only 0.56 g/L vanillin was produced with the molar yield of 47.67% at 40 °C (Fig. 4a). This indicated that the production of vanillin from ferulic acid using *B. subtilis* immobilized on CFT carrier was not always enhanced at elevated temperatures. Previous study also revealed that EPS production and biofilm formation did not take place at extreme temperature but did so at intermediary temperature^20^.

It is well known that the system pH plays a crucial role in biotransformation. Generally, variation in pH can affect both the ionic state of substrate and enzymes involved in the biochemical reaction^21^. To verify the effect of pH on the biotransformation, experiments were carried out at pH ranging from 8.0 to 9.5. The HRT, temperature, stirring speed, and ferulic acid concentration were kept at 20 h, 35 °C, 200 rpm, and 1.5 g/L, respectively.

As shown in Fig. 4b, increasing the pH from 8.0 to 8.5 led to enhance the production of vanillin, a maximum molar yield of 54.73% was obtained with 0.64 g/L vanillin at pH of 8.5. *Bacillus* species can survive in a wide range of pH 6.0–12.0 but they need an alkaline pH of 8.0–10.0 for growth and enzyme secretion^22^. The negative charge density on the surface of cells would increase with the increase of pH, resulting in a tight cell attachment on CFT carrier^23^. Additionally, as the pH increased, the solubility of ferulic acid increased and more ferulic acid molecules become negatively charged due to its low pKa of 4.8^24^.

However, as is evident from the figure, pH higher than 8.5 tended to have negative effect on the vanillin production. The vanillin molar yield at pH 8.5 declined to 53.5% at pH 9.0 and 53.1% at pH 9.5, respectively (Fig. 4b). It has been stated that the optimal pH for the activity of protease from *B. subtilis* was between 8.5 and 9.0^25^. Increasing pH led to the reduction of activity of enzyme involved in biotransformation. Furthermore, high pH usually results in reducing the production of EPS which have an important effect on biofilm formation^26^. In reactor, biofilm formation on CFT carrier is not an isolated phenomenon, but an equilibrium process between the planktonic and sessile states of bacteria^27^. Differences in the physicochemical properties of planktonic cells at different pH levels would change their cell wall composition, which could have resulted in negative effect on the equilibrium biofilm thickness. Moreover, the increase in electrostatic repulsion between the cell and ferulic acid due to the increase of pH might lead to the decrease of vanillin production.

Oxygen is an indispensable raw material in aerobic fermentation. Oxygen levels could affect the transcription and/or synthesis of different enzymes, resulting in the changes of cell metabolism, product yield and productivity^28^. The supply of oxygen can be achieved by means of mechanical stirring. Generally, aerobes including *B. subtilis* require large amounts of oxygen for NAD(P)H or FADH2 reoxidization and ATP generation during cell growth and proliferation^29^. It has been stated that stirring speed was an important factor for *B. subtilis* and its products production in bioreactor^30^. In order to determine the effect of stirring speed on biotransformation, the reactor was operated at a HRT of 20 h with temperature of 35 °C, initial pH of 9.0, and initial ferulic acid concentration of 1.5 g/L. The stirring speed was varied from 150 to 300 rpm.

**Figure 5:**
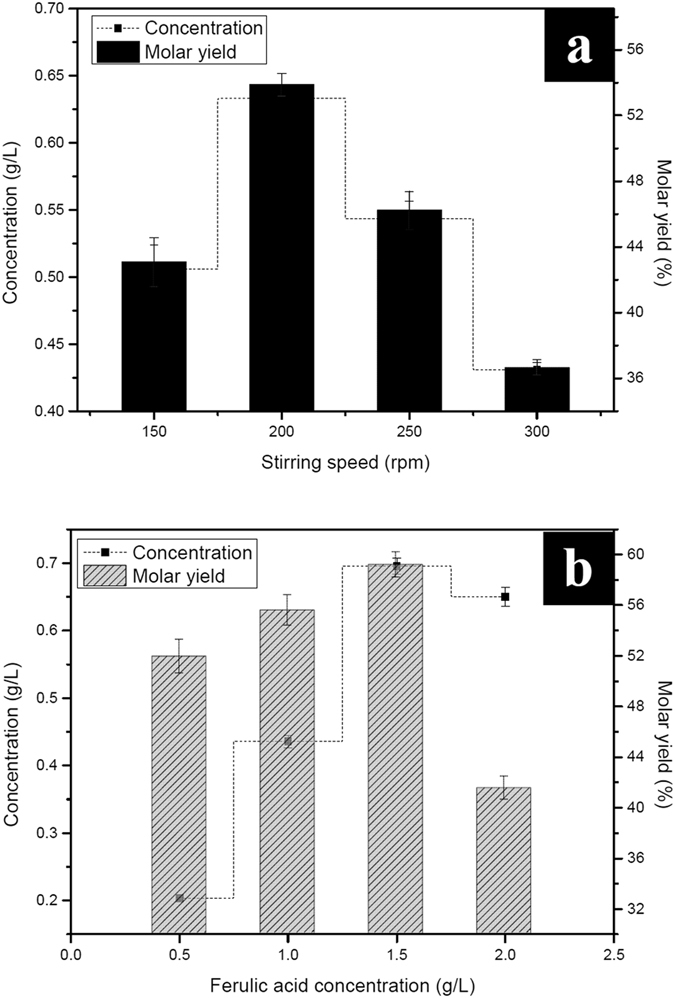
Effect of stirring speed (**a**) and initial ferulic acid concentration (**b**) on the production of vanillin.

It can be seen form Fig. 5a that increased the stirring speed from 150 to 200 rpm resulted in an increase in vanillin production. The vanillin yield peaked quickly. Based on the maximum vanillin production at 0.63 g/L with a molar yield of 53.86%, 200 rpm was found to be the optimum speed. This indicated that the stirring speed less than 200 rpm did not benefit the production of vanillin. The explanation was related to the oxygen transfer rate in the reactor. The stirring speed was closely related to the oxygen partial pressure which has a major effect on the oxygen solubility and the mass transfer driving force in the liquid^31^. At lower stirring speed, the oxygen partial pressure was lower and led to a low oxygen solubility^32^. Hence, the vanillin yield is lower. Previous literature revealed that low stirring speed can cause a drastic reduction in the protease production by *Bacillus* sp^32^.

When the stirring speed increased, the oxygen solubility was higher and resulted in a higher oxidative capacity of the broth. Surprisingly, the vanillin production did not increase and was found to be negatively influenced by variations in stirring speed beyond 200 rpm (Fig. 5a). Stirring is crucial for better oxygen and nutrient transfer during biotransformation. However, very high dissolved oxygen could hamper the production of the target product due to the suppression of enzyme activities required in producing target product^28^. Meanwhile, high dissolved oxygen concentration would result in the oxidization of vanillin to further products^1^. Therefore, the overall vanillin yield decreased. Additionally, higher stirring speed might cause shear stress on bacterial cells resulting in the reduction of biomass^32^. Furthermore, stirring might change the hydrodynamic condition which was defined as the transport of cells, nutrients, and oxygen from the fluid to the biofilm. The hydrodynamic condition could affect the structure, physiological composition and metabolic characteristics of biofilm^33^. High stirring speed would lead to the reduction of biofilm thickness, volumetric density and cell density, resulting in a decrease of vanillin yield. Moreover, rapid stirring could cause foam in *B. subtilis* fermentations, which led massive overflow of broth^30^. Power consumption during stirring is a significant fraction of the total operating cost. Hence, increasing the stirring speed would increase product cost.

Increasing substrate concentration required to obtain high-yield of product so that the process is profitable. The following experiments were aimed at evaluating the effect of initial ferulic acid concentration on biotransformation. Experiments were carried out at pH 9.0, HRT 20 h, temperature 35 °C, stirring speed 200 rpm, and different ferulic acid concentration: 0.5, 1.0, 1.5 and 2.0 g/L. It can be clearly observed from Fig. 5b that 0.5 g/L of initial ferulic acid concentration resulted 0.20 g/L of vanillin together with a molar yield of 51.93%. Both the vanillin molar yield and the total amount of vanillin increased correspondingly with increasing initial concentration of ferulic acid. A maximum yield of 0.70 g/L vanillin with molar yield of 59.20% appeared at initial concentration of 1.5 g/L (Fig. 5b). However, the vanillin production declined if initial ferulic acid concentration exceeded 1.5 g/L.

These revealed that ferulic acid at a concentration under 1.5 g/L could have no adverse effect, whereas a higher ferulic acid concentration might negatively affected biotransformation. It has been stated that high concentrations of ferulic acid were toxic for microbial cells and could inhibit the growth of microbes^2^. In this study, a ferulic acid concentration higher than 1.5 g/L might inhibit the growth of planktonic cell, which in turn led to the break of equilibrium process between the planktonic and sessile states of bacteria during biofilm formation^27^. Higher concentration of ferulic acid also led to an increase in viscosity of the fermentation broth, which in turn negatively influenced the volumetric oxygen mass transfer, ultimately resulting in lower yield of protease and EPS^32^. Thus, low protease production rates would lead to a limited enzymatic biotransformation. Meanwhile, the reduction of EPS could not only impair the structural integrity of the biofilm, but lessen the nutrient reserve which ensure cell survival under famine conditions^34^, resulting in low vanillin production.

**Figure 6:**
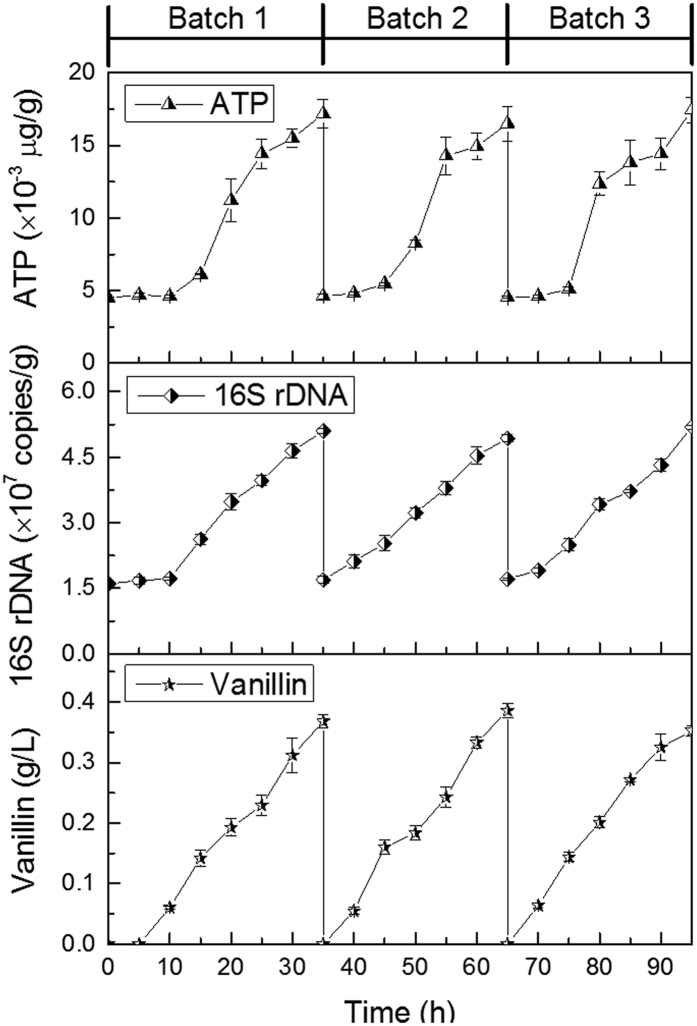
Time profiles of vanillin concentration, 16S rDNA copy number and ATP content in the repeated batch biotransformation in the stirring reactor inoculated *B. subtilis* without CFT carrier (free cells).

To understand the time course of biotransformation by free and immobilized cells of *B. subtilis*, the repeated batch process was performed varying the length of each batch in function of maximum vanillin production. Figure 6 depicts the results of the repeated batch biotransformation carried out in the stirring reactor using free cells. In batch 1, fermentation exhibited a lag phase of 10 h, which was longer than that of the two subsequent batches (5 h) (Fig. 6). The shortened lag phase was because the cells from batch 1 were reused to inoculate the next batch^35^. After 35 h fermentation, a maximum vanillin titer of 0.37 g/L was obtained. The cell growth and the cellar activity increased continuously during biotransformation. The maximum values for 16S rDNA copy number and ATP concentration were 5.10 × 10^7^ copies/g and 0.017 μg/g at 35 h, respectively (Fig. 6). The fermentation time for batch 2 and 3 was the same, both were 30 h. A gradual increase of cell growth and cellar activity were observed in batch 2 and 3. Regarding vanillin production, similar values obtained in batch 2 and 3, in the range of 0.35–0.38 g/L, were achieved (Fig. 6).

**Figure 7:**
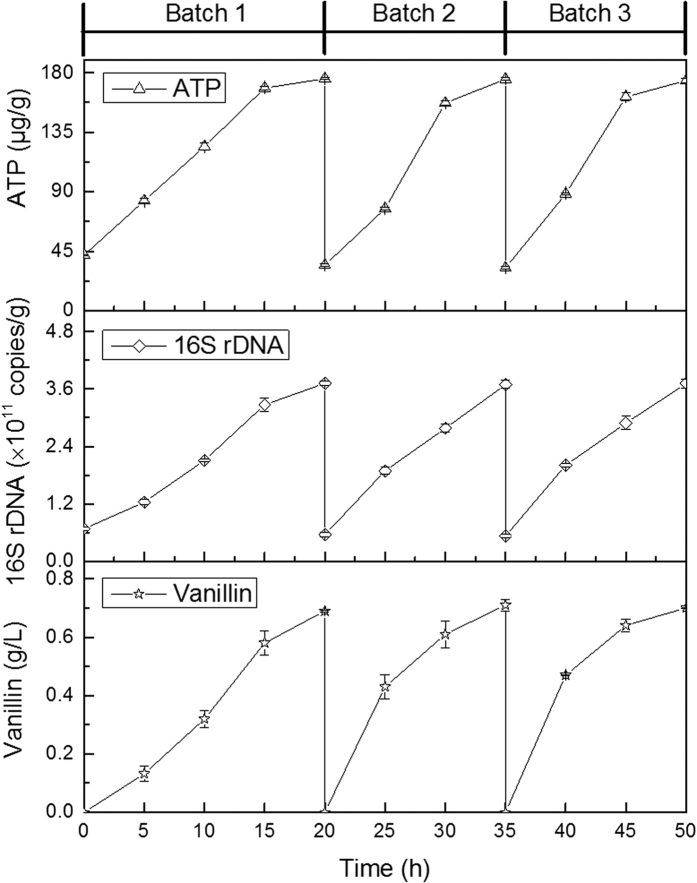
Time profiles of vanillin concentration, 16S rDNA copy number and ATP content in the repeated batch biotransformation in the stirring fixed-CFT reactor immobilized *B. subtilis* (immobilized cells).

Figure 7 reports the repeated batch biotransformation carried out using immobilized *B. subtilis* cells under the optimized conditions. It can be found that the sustainable continuous increases of cell growth and cellar activity among the three experiments (repeated batch 1–3) occurred on the production of vanillin (Fig. 7). The lag phase of batch 1 was about 5 h, and was eliminated in batch 2 and 3. Consequently, the fermentation time of batch 2 and 3 was only 15 h. Similar values of 16S rDNA copy number obtained in all batches, ranging from 3.69 × 10^11^ to 3.72 × 10^11^ copies/g, were achieved (Fig. 7). The concentration of ATP obtained in batch 2 and 3 was the same, or higher, than that of batch 1. It also can be seen that the vanillin titer of each repeated batch cycle was more or less constant, ranging from 0.68 to 0.71 g/L (Fig. 7).

During repeated batch biotransformation, the cell growth and cellar activity in liquid were also detected. The copy numbers of 16S rDNA varied in a small range of 4.17 × 10^6^–3.12 × 10^7^ copies/g, maintaining relatively stable throughout the repeated batch period. Accordingly, similar values of ATP titer in the range of 0.004–0.022 μg/g, were achieved in the three experiments. These revealed that very little amount of free cells (approximately 10^−5^–10^−6^ fold less than the values for immobilized cells) grew during each batch fermentation in the stirring fixed-CFT reactor immobilized *B. subtilis*, and probably its contribute to the biotransformation was minimal. After stopping fermentation, the biomass was obtained from liquid sample of the immobilized cell system, and its concentrations for batch 1, 2 and 3 were 0.29, 0.32 and 0.34 g/L, respectively. The low values of free biomass allowed easy transfer operations from a batch to the following and vanillin recovery^36^. In order to understand the adsorption of vanillin to biomass, the vanillin in the biomass precipitate was analyzed. Results showed that vanillin titer obtained from the three batch biotransformation was the same or lower than 0.004 g/L. This indicated that vanillin adsorbed to cell biomass (ca. 1.1%) was very scare and its effects on vanillin titer of liquid sample were negligible.

Comparing Fig. 6 with Fig. 7, the fermentation period of the immobilized cell system was shorter than that of free cell system. This was because that the immobilized cell system only required as a one-off seed culture, which would shorten the fermentation period significantly^37^. It was evident that immobilized cell system was able to obtain maximum vanillin production after only 15 h while the free cells needed double time (30 h). It is worth mentioning that both 16S rDNA copy number and cellar activity in the immobilized cell system were higher than those from the free cell system. The reason was because the CFT carrier act as a protection barrier for access of the inhabited factors to the cell^38^. The vanillin molar yield (58.70%, 60.43% and 59.59% for batch 1, 2 and 3, respectively) and productivity (0.034, 0.047 and 0.046 g/L/h for batch 1, 2 and 3, respectively) from the immobilized cell system were higher than those from the free cell system (31.30%, 32.81% and 30.00% of molar yield for batch 1, 2 and 3, respectively; 0.011, 0.013 and 0.012 g/L/h of productivity for batch 1, 2 and 3, respectively). This might be attributed to the synergistic effect of cell reuse and microorganism acclimation^35^.

To date, several studies have been carried out to biotechnologically produce vanillin from ferulic acid using various organisms^1^,^2^,^39^,^40^,^41^,^42^,^43^,^44^,^45^,^46^,^47^,^48^. The reported maximum vanillin productivity and molar yield obtained using free mutant *Pseudomonas putida*, engineered *Pseudomonas fluorescens*, recombinant *Escherichia coli* were much higher than those of *B. subtilis* B7-S in this study (Table 1). Although the production of vanillin can be increased through genetic manipulation of these bacteria mentioned above, the cost of these methods could be too high due to the complex operation^49^. Additionally, the genetic instability of these recombinant strains might occur during biotransformation^2^. The maximum vanillin productivities for free *Amycolatopsis* sp. HR167, *Streptomyces* sp. V-1 reported by previous studies reached 0.061 and 0.34 g/L/h, respectively (Table 1), which much higher than 0.047 g/L/h in this work. However, by the immobilized cell strategy here, the maximum molar yield is at least 1.11 fold higher than that of free cells mentioned above. This suggested that the stirring bioreactor packed with CFT carrier biofilm formed by *B. subtilis* could efficiently transform ferulic acid to vanillin. Although the reported maximum molar yield obtained using free *Streptomyces halstedii* GE 107678 and mix culture of *Aspergillus niger* and *Pycnoporus cinnabarinusi* was higher than that of immobilized *B. subtilis* B7-S in this study, the immobilized cell system could shorten the fermentation time and obtain a higher value of the maximum vanillin productivity (Table 1). The current work was most effective in producing vanillin (immobilized repeated batch system) compared to the closest counterpart (immobilized batch or fed-batch system) in Table 1. Additionally, the immobilized cell system reached the highest vanillin production as compared to previous fermentations carried using frees of *B. subtilis*^45^. Therefore, the reactor packed with CFT biofilm of *B. subtilis* in this study is a stable and highly efficient process to produce vanillin from ferulic acid.

In conclusion, biotransformation of ferulic acid to vanillin by *B. subtilis* in a stirring packed-bed reactor filled with CFT was investigated. Results obtained from SEM, HPLC, qRT-PCR and ATP assay during immobilizing indicated that *B. subtilis* could be successfully immobilized and form biofilm on the surface of CFT carrier, and biotransformation was closely related with the extent of biofilm formation. Among parameters relating to biotransformation, temperature, stirring speed and initial ferulic acid concentration were found to have a significant influence on vanillin production. Repeated batch biotransformation indicated the maximum vanillin molar yield (60.43%) and productivity (0.047 g/L/h) from the immobilized cell system was higher than those from the free cell system (molar yield of 32.81%, productivity of 0.013 g/L/h). These results revealed that the reactor packed with CFT biofilm of *B. subtilis* possesses a good application potential in producing vanillin from ferulic acid.

## Methods

### Chemicals

Vanillin standard was purchased from Sigma-Aldrich (Shanghai, China). Analytical-grade ferulic acid was obtained from Aladdin (Shanghai, China). Solvents used in high-performance liquid chromatography (HPLC) analysis were of HPLC grade. Other chemicals were of analytical grade.

### Bacterium and medium

*B. subtilis* CCTCC M2011162 was used for the biotransformation of ferulic acid. It has been proved that this strain is able to transform ferulic acid to vanillin with relatively high yield of vanillin^50^. Seed medium (pH 8.0) contained 5 g/L glucose, 10 g/L peptone, 3 g/L yeast extract powder, and 5 g/L NaCl. Immobilized medium (pH 8.0) contained 10 g/L peptone, 3 g/L yeast extract powder, 5 g/L NaCl and 0.5 g/L ferulic acid. Biotransformation medium (pH 8.0–9.5) contained 10 g/L peptone, 3 g/L yeast extract powder, 5 g/L NaCl and 0.5–2.0 g/L ferulic acid. The pH of media mentioned-above were adjusted by 5 M NaOH.

### Bioreactor and cell immobilization

**Figure 8:**
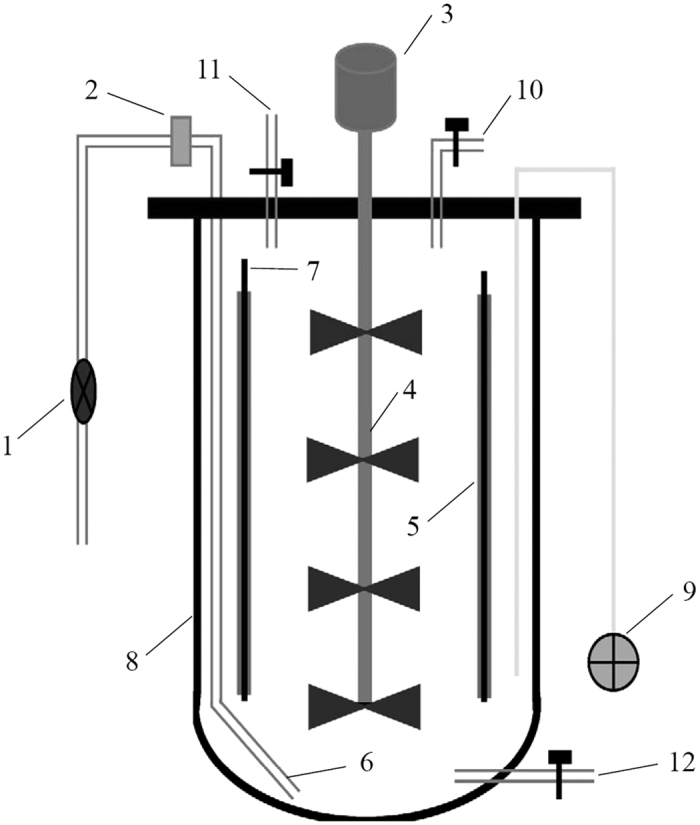
Schematic diagram of the packed bed-stirred fermentor (1. air flow meter, 2. air filter, 3. motor, 4. stirrer, 5. carbon fiber textiles, 6. air inlet, 7. stainless steel support, 8. tank body, 9. temperature controller, 10. air outlet, 11. feed inlet, 12. discharge port).

The stirred stainless steel fermenters packed with active carbon fiber (Xintong Activated Carbon Fiber Co., Ltd., Nantong, China) as the biofilm carrier were used in this study (Fig. 8). The fermenter has an effective volume of 10 L (21 cm outside diameter and 30 cm height). A frustum of a cone composed of eight stainless steel wires and two rings was laterally packed by CFT. This conical frustum (12 cm top diameter, 16 cm bottom diameter, and 25 cm height) was placed into the reactor as biofilm carriers.

The reactor containing 7.6 L of immobilized medium without ferulic acid was steam sterilized. Then the filtered ferulic acid solution (dissolved in 1 M NaOH solution, pH 8.0) was directly added to the rector. The *B. subtilis* was cultured in seed medium. After reaching stationary growth phase, 400 mL of cultures were pumped by a peristaltic pump into the reactor with immobilized medium. The immobilization was performed in this rector at 150 rpm and 35 °C for about 40 h. An air pump with 0.22 μm filter was used to provide sufficient air and ensure the system was never oxygen limited (Fig. 8). Approximately 20 mL liquid sample and 4 cm^2^ CFT biofilm carrier sample were taken at 10 h intervals during immobilizing. The extent of biofilm formation, cell growth, cellular activity, and vanillin concentration were detected.

### Optimization of reaction parameters

Bioconversion experiments were carried out in the immobilized reactor described above. The sterile biotransformation media without ferulic acid and the filtered ferulic acid solution were pumped into the reactors. In order to obtain the maximum yield of vanillin in the stirring fixed-CFT reactor immobilized with *B. subtilis*, different HRTs (10–40 h), temperature (25–40 °C), initial pH (8.0–9.5), stirring speed (150–300 rpm), and ferulic acid concentration (0.5–2.0 g/L) were evaluated in this study. Approximately 20 mL liquid sample was withdrawn at preestablished intervals for vanillin determination.

### Repeated batch biotransformation

Biotransformation were conducted both in the stirring fixed-CFT reactor immobilized *B. subtilis* (immobilized cells) and in the stirring reactor inoculated *B. subtilis* without CFT carrier (free cells) under the optimized condition obtained by the above-mentioned single factor experiments. Repeated batch biotransformation were performed removing the exhausted medium at the time of maximum vanillin production and, after washing the CFT carrier to remove free cells and residue twice with sterile distilled water, adding 8 L fresh biotransformation medium. Repeated batch operations were carried out with three cycles. For each biotransformation run, approximately 20 mL liquid sample and 4 cm^2^ CFT biofilm carrier sample were withdraw every 5 h for vanillin analysis, cell growth determination, and cellular activity detection. After stopping each batch biotransformation, the liquid sample obtained from the immobilized cell systems were centrifuged for biomass measurement and vanillin analysis.

### Analysis

The concentration of vanillin was determined by HPLC as described in (Information S1 in the Electronic Supplementary Materials). Vanillin molar yield was calculated as the ratio between the produced vanillin (mM) and the initial ferulic acid (mM)^2^. The extent of biofilm formation were examined by a scanning electron microscope (EVO-18, Carl Zeiss, Germany). The sample was cut from the carriers and dried naturally. All samples were mounted and sputtered together to ensure there should be no difference in coating thickness. Bacterial cell growth was measured using real-time fluorescent quantitative PCR as described in (Information S2 in the Electronic Supplementary Materials). The ATP content was used as an indicator to evaluate the cellular activity of *B. subtilis* in different systems. It was measured using an ATP assay kit (Beyotime, Nanjing, China) according to the manufacturer’s instructions based on the bioluminescence technique (Information S3 in the Electronic Supplementary Materials). Biomass was measured using dry weight method reported by previous study^51^. All experiments were carried out in triplicate and the average values were reported. A *t*-test was used to compare the batch to batch variation using SPSS 19 statistical software (SPSS Inc., Chicago, USA).

## Additional Information

**How to cite this article**: Yan, L. *et al*. Biotransformation of ferulic acid to vanillin in the packed bed-stirred fermentors. *Sci. Rep*. **6**, 34644; doi: 10.1038/srep34644 (2016).

## Supplementary Material

Supplementary Information

## Figures and Tables

**Table 1:** Production of vanillin from ferulic acid by various organisms.

Organisms	Fermentation technique	Maximum productivity (g/L/h)	Maximum molar yield (%)	Reference
*Amycolatopsis* sp. HR167	Batch (free cell)	0.061	50.98	44
Mutant *Pseudomonas putida*		0.052	73.00	1
*Aspergillus niger* and *Pycnoporus cinnabarinus*		0.038	61.90	47
*Pycnoporus cinnabarinus* MUCL 39533		0.0021	22.00	1
*Pseudomonas* sp. EF1		0.0035	47.00	1
*Streptomyces halstedii* GE 107678		0.0062	80.00	40
*Staphylococcus aureus*		0.0019	59.20	43
*Pycnoporous cinnabarinus*		0.0008	54.00	46
Engineered *Pseudomonas fluorescens*		0.053	84.10	2
Recombinant *Escherichia coli*		0.21	86.60	41
*B. subtilis* B7-S	Repeated batch (free cell)	0.028	18.42	45
*B. subtilis* B7		0.021	13.33	45
*Haematococcus pluvialis*	Fed-batch (immobilized cell)	0.0001	6.97	39
*Streptomyces* sp. V-1	Fed-batch (free cell)	0.34	54.50	39
*Capsicum frutescens*	Batch (immobilized cell)	0.00002	2.07	48
*B. subtilis* B7-S	Repeated batch (immobilized cell)	0.047	60.43	This study
