# Supplementary material for: Biotransformation of ferulic acid to vanillin in the packed bed-stirred fermentors
Source: Sci Rep. 2016 Oct 6;6:34644. doi: 10.1038/srep34644 (PMC5052561; doi:10.1038/srep34644)
Supplement: Supplementary Information [file srep34644-s1.doc]

**Biotransformation of** **ferulic acid to vanillin in the** **packed bed-stirred fermentors**

Lei Yan ^a, †^, Peng Chen ^b, †^, Shuang Zhang ^a^, Suyue Li ^c^, Xiaojuan Yan ^c^, Ningbo Wang ^c^, Ning Liang ^c^, Hongyu Li b, [[1]](#footnote-2)*

**Electronic Supplementary Materials**

**Information S1:** HPLC analysis of vanillin

HPLC analysis was performed on a HPLC system (LC-2010A, Shimadzu, Japan) equipped with a reverse-phase C18 column (5 μm, 250 mm × 4.6 mm) (Elite, Dalian, China) and a UV–Vis detector (280 nm). The injection volume was 20 μL, the mobile phase consisted of methanol and water (40:60, v/v), and the flow rate was 0.8 mL/min, and the column temperature was maintained at 25 °C. Two milliliter of liquid sample or 1 mg centrifuged sample was added into 8 ml methanol solution (50%, v/v) and mixed throughoutly. The mixture was centrifuged at 3000 rpm for 5 min and then filtered using 0.22 μm polytetrafluoroethylene membrane. The filtrate was used for HPLC analysis.

**Information S2:** Quantitative RT–PCR analysis

Total DNA was extracted from 1 g (wet weight) of CFT carrier or liquid culture sample using a Bacterial Genomic DNA Extraction Kit (Biomed, Beijing, China) according to the manufacturer’s protocol.

The qRT-PCR were performed in 96-well blocks with a Bio-Rad CFX-96 real time system (Bio-Rad, Hercules, California, USA). The bacterial 16S rRNA gene was quantified using specific primers for *B. subtilis* (upstream primer 5′-ACGAGCGCAACCCTTGA-3′, downstream primer 5′-ACGTCATCCCCACCTTCCT-3′) [^1^](#_ENREF_1). Each reaction mixture included 12.5 μL of 2×SYBR Premix Ex Taq™ (Takara, Dalian, China), 0.5 μL of 50×ROX™ Reference DyeII (Takara, Dalian, China), 0.5 μL for each primer (10 μM), 2 μL of template DNA, and sterilized distilled water to make a final volume of 25 μL. The thermocycling steps were as follows: initial denaturation step (4 min at 95 ^o^C), 40 cycles of denaturation (30 s at 95 ^o^C), annealing (45 s at 60 ^o^C) and elongation (35 s at 72 ^o^C), and a final extension step (7 min at 72 ^o^C). The copy numbers of the standard plasmid insert of the 16S rRNA gene of *B. subtilis* ranged from 6.7×10^1^ to 6.7×10^5^ copies/µL. Each qRT-PCR experiment was conducted in triplicate. The amplification efficiency (E) and correlation coefficients (R^2^) of the primers were 0.996 and 109.1%, respectively.

**Information S3:** ATP analysis

The biofilm sample was scraped and washed from 1 g (wet weight) of CFT carrier. The suspension of biofilm sample or 1 g liquid culture sample was centrifuged at 12,000 rpm for 5 min. Two hundred microliters of lysis buffer was added to lyse the pellet cells and vibrated in order to insure the cells were completely split. After centrifuged at 12,000 rpm for 5 min at 4 °C, 100 μL of the supernatant of each sample was transferred to a 96-well plate together with 100 μL ATP detection buffer. The bioluminescence was assayed using an Infinite M200 PRO microplate reader (Tecan, Männedorf, Switzerland). The protein content (BCA Protein Assay kit, Beyotime, Nanjing, China) was used to correct the different samples for comparison.

**Reference**

1. Pan K., Feng X., Cui H., Zhang Y., Zhao S. Detecting the variation intestinal Bacillus by real-time fluorescence quantification PCR after chick ora*l Bacillus subtil*i*s Tenth Session of the Fourth Academic National Symposium and Development & Strategy Forum for Animal Microecological Enterpri*se 2010; 2:688-693.

1. ^^† The author contributed equally to the manuscript.

   ^*^ Corresponding author. Tel. & Fax: +86 931 8915686.

   E-mail address: hekouyanlei@gmail.com (L. Yan), chenpeng@lzu.edu.cn (P. Chen). [↑](#footnote-ref-2)
